# Th17, intestinal microbiota and the abnormal immune response in the pathogenesis of celiac disease 

**Published:** 2015

**Authors:** Clelia Cicerone, Raffaella Nenna, Stefano Pontone

**Affiliations:** 1 *Department of Internal Medicine and Medical Specialties, “Sapienza” University of Rome, Italy*; 2 *Department of Pediatrics, “Sapienza” University of Rome, Italy *; 3 *Department of Surgical Sciences, “Sapienza” University of Rome, Italy *

**Keywords:** Celiac disease, Immune response, HLA, Interleukin, Microbiota

## Abstract

Celiac disease (CD) is an autoimmune enteropathy induced by the ingestion of gluten in genetically predisposed individuals who carry the HLA-DQ2 or -DQ8 alleles. The immune response is abnormal in celiac disease with small intestinal epithelial damage via CD8+CD4- intraepithelial lymphocytes. The etiology is multifactorial involving genetic and environmental factors, an abnormal immune response, and intestinal dysbiosis. The innate and acquired T-cell mediated immunity play important roles in the pathogenesis of this disease, particularly CD4+ Th17 cells, which have been shown to have critical functions in host defense against bacterial pathogens and in the inflammatory responses to deamidated gluten peptides. We review what is known about the interaction between immune system and intestinal microbiota in the pathogenesis of celiac disease.

## Introduction

 Celiac disease (CD) is a chronic autoimmune disorder induced by the ingestion of gluten proteins of wheat (gliadins) and other related cereals (rye and barley) proteins (hordeins and secalins) in genetically predisposed individuals (1). This autoimmune disorder affects the small bowel and often produces symptoms as diarrhea, malabsorption, and extraintestinal symptoms (1). The HLA locus is the main genetic influence. For example the HLA-DQA1*05:01 and DQB1*02:01 alleles forming the particular DQ2.5 haplotype confer high susceptibility to CD (2). A recent study also reveals that the HLA-DQ2 genotype strongly influences the intestinal colonization of infants at family risk of developing CD. Infants with high genetic risk of developing the disease (HLA-DQ2 carriers) show reduced abundance of Actinobacteria (Bifidobacterium species) and increased abundance of Firmicutes (3). The typical histological features of CD include atrophy of the small intestinal villi, hyperplasia of the crypts, and a marked infiltration of the *lamina propria* and intraepithelial compartments with inflammatory cells (4). 

The prevalence of CD in adults in the United States ranges from 0.7% to 2.3%, and 1.3% in Italian school-age children (5). In addition, several studies have shown that, despite a prevalence comparable to those of European nations, CD remains underdiagnosed in the United States (6,7).

The only treatment for CD is lifelong adherence to a gluten-free diet. It has been demonstrated to improve the symptoms, reduce the risk of malignancy, and impart other health benefits such as an improvement in bone mineral density (8,9). 

Recently, oats have been receiving increasing interest as food to celiac patients. The incorporation of oats into a gluten-free diet provides high fiber and vitamin B. However, it is recommended that individuals with CD should have both initial and long-term assessments by a health professional when introducing pure oats into a gluten-free diet (10). Real A et al., indicates that some cultivars of oats can be a safe part of a gluten free diet suggesting that there is wide range of variation of potential immunotoxicity of oat cultivars (11).

## Immunopathology of celiac disease and role of IL-17

It is well known that innate and acquired T-cell mediated immunity play important roles in the pathogenesis of the disease. In CD, the T-cell mediated adaptive response is mediated by CD4+ Th1 lymphocytes in the lamina propria. Deamidated gluten peptides are presented to CD4+ Th cells with subsequent release of inflammatory cytokines. We know that the lamina propria of the small intestine contains large numbers of two homeostatically regulated and developmentally related populations of CD4 T cells, IL-17+ helper Th17 cells and Foxp3+ regulatory T cells (Treg) (14-15). Th17 cells produce the cytokines IL-17 (also known as IL-17A), IL-17F, and IL-22. Among these, IL-17 has been the most thoroughly studied and is considered the signature effector cytokine for this subset. In humans and mice, the IL-17 cytokine family consists of six members: IL-17A (also referred to as IL-17), IL-17B, IL-17C, IL-17D, IL-17E (also known as IL-25) and IL-17F (16-18). 

Similar to Th1 or Th2 cells, in vivo, differentiation of naıve CD4+ T cells into Th17 cells requires T cell receptor recognition of its cognate antigen presented on major histocompatibility complex (MHC) class II by professional antigen-presenting cells (APCs), such as dendritic cells (Dcs). 

 Th17 cell differentiation in vitro from naïve T cells requires furthermore, the coordinated action of multiple cytokines including TGF-β (17-20) and also has been proved in vivo, that certain bacterial species are potent immune stimulators (21,22).

Functionally, IL-17A participates in inflammatory responses inducing neutrophil granulopoiesis by stimulating epithelial cells to secrete granulocyte colony-stimulating factor (G-CSF). Furthermore, IL-17A and IL-17F can directly recruit and activate neutrophil cellular responses at sites of inflammation (14). Given the expanding roles of IL-17A and IL-22 in mediating innate barrier responses, it is not surprising that the IL-17A IL-22 axis is emerging as a central element of mucosal immunity to microbial challenge. In fact, Th17 cells have been shown to have critical functions in host defense against bacterial and fungal pathogens, particularly those encountered at mucosal surfaces (23-25). Recent studies confirmed that mucosal IL-17A response was elevated at the late stage of CD when villous atrophy has developed. Mucosal IL-17A displayed elevated expression in children with untreated CD when compared to GFD-treated children and children with potential CD (26-29).

Another study revealed elevated interleukin IL-17 responses after exposure to wheat gliadin in acute CD, but not in potential CD, thus indicating the association of upregulated IL-17 pathway with villous atrophy. However, T-cell clones reactive with deamidated gliadin peptide did not show IL-17 secretion, which suggests that activation of IL-17 may not be induced directly by dietary gluten but rather develops at later stage of mucosal inflammation (30,31).

IL-17 in mucosal antimicrobial defense has been shown to contribute to the gut barrier function and upregulation of IL-17 decrease the dissemination of pathogens from the intestinal lumen (32). On the other hand, commensal bacteria can induce mucosal IL-17 response (33-34) and it is possible that changes in microbiota could be responsible for the upregulation of IL-17 when villous atrophy develops.

The cytokine profile can be different in patients with refractory CD (RCD) or active CD. In the RCD the symptoms/signs of malabsorption and villous atrophy persist or recur despite a strict GFD for more than 12 months and in the absence of other disorders (35).

A recent study (36) have analyzed on duodenal biopsies, inflammatory cytokines by real-time PCR and ELISA in patients with RDC, and active CD. IFN (interferon)-γ and IL (interleukin)-21 transcripts were increased in active CD patients but not in RCD patients as compared to normal controls, whereas IL-17A RNA was upregulated in both active CD and RCD. The findings indicate that the profile of mucosal effector cytokines differs between RCD and active CD and suggest that TNF-α, IL-6 and IL-17A, but not Th1-type cytokines, could drive the detrimental response in this condition.

## Intestinal microbiota, immune system and celiac disease

The composition of intestinal microbiota and intestinal dysbiosis has been implicated in the pathogenesis of celiac disease (36,37). The intestinal microbiota is the collection of microbes that reside in the gastrointestinal (GI) tract and is comprised of over 1000 different species that contributes 3.3 million unique microbial genes in the GI tract of humans (38,39). This intricate microbial system includes bacteria, which live in a symbiotic relationship with their host, and some microbes, which have potentially pathogenic characteristics. There are four dominant phyla: Firmicutes, Bacteroidetes, Actinobacteria and Proteobacteria (38). Firmicutes and Bacteroidetes account for >90% of the bacterial population in the colon while Actinobacteria and Proteobacteria (which includes Enterobacteriaceae) are regularly present but are scarce (<1%–5%) (36).

Molecular techniques have shown that, compared to the fecal and duodenal microbiota of healthy individuals, the fecal and duodenal microbiota of CD patients is characterized by the presence of higher numbers of gram-negative bacteria (bacteroides and enterobacteria) and lower numbers of gram-positive bacteria, like bifidobacteria. The differences between active and non-active CD seem to be associated with a decreased abundance of members of the family Streptococcaceae, specifically the S. anginosus and S. mutans groups. The active phase of the disease was also associated with increased proportions of Enterobacteriaceae and Staphylococcaceae and, in particular, the species Klebsiella oxytoca, S. epidermidis, and S. pasteuri. (37,40).

**Figure 1:**
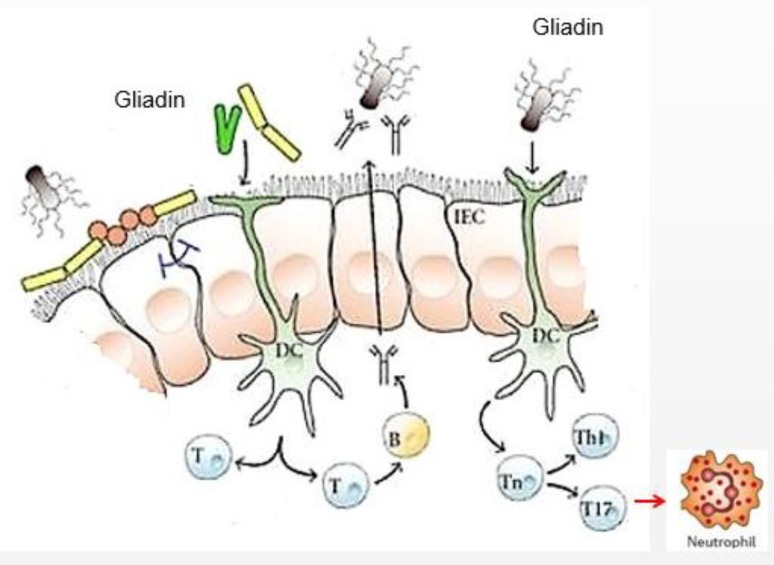
Expansion of B and T cells in Peyer’s patches in response to gliadin.

These alterations are attenuated after long-term adherence to a gluten-free diet, but the microbiota is not completely restored; in particular, a reduced abundance of specific species of Streptococcus (S. anginosus and S. mutans) also characterizes the microbiota of CD patients with active and non-active disease. A recent study (41) showed that the treated celiac disease patients with persistent symptoms were colonized by different duodenal microbiota in comparison with patients without symptoms. The treated patients with persistent symptoms had a higher relative abundance of Proteobacteria (p=0.04) and a lower abundance of Bacteroidetes (p=0.01) and Firmicutes (p=0.05). The intestinal microbiota plays a crucial role in the development of local and systemic immunity, as well as in maintaining colonic homeostasis (42,43). For example, to drive the expansion of B and T cells in Peyer’s patches and mesenteric lymph nodes, especially CD4+ T cells (Fig.1) (44).

Besides segmented filamentous bacteria, which adhere closely to the intestinal epithelium, have been shown to induce Th17 responses (32) and increase the number of Treg cells in both the small intestine and colon (45).

Although IL-17A and IL-22 secretion can be a hallmark of the adaptive phase response to infection, it is becoming increasingly clear that bacterial pathogens trigger rapid IL-17A- and IL-22-dependent innate defense in the gut mucosa (46). Moreover, the production of these cytokines in the intestine seems to be regulated at the homeostatic level by the interaction between the host and the intestinal microbiota. In a recent study, Jose´ Moise´s Laparra and colleagues have demonstrated that the administration of Bifidobacterium longum CECT 7347, previously selected for reducing gliadin immunotoxic effects in vitro, could exert protective effects in an animal model of gliadin-induced enteropathy by increasing NFkB expression and IL-10, but reducing TNF-a production. In sensitized gliadin-fed animals, CD4+, CD4+/Foxp3+ and CD8+ T cells increased, whereas the administration of B. longum CECT 7347 reduced CD4+ and CD4+/Foxp3+ cell populations and increased CD8+ T cell populations (47). These results confirmed and another study (48) that showed that Bifidobacterium strains with immunoregulatory properties have been shown to suppress the pro-inflammatory cytokine pattern induced by the altered colonic microbiota of CD patients.

## Conclusions

In conclusions this review suggests that the IL-17A producing cells play a major role in the pathogenesis of CD, and that both gluten and bacteria provoke an IL-17A response in the intestinal mucosa of CD patients. The upregulation of IL-17 immunity is associated with untreated CD and especially villous atrophy, whereas mucosal IL-17 immunity is not present in potential or GFD treated CD. IL-17 is a marker of active CD and its role as a predictive biomarker of villous atrophy should be evaluated.
